# Trends in the contemporary incidence of colorectal cancer and patient characteristics in the United Kingdom: a population-based cohort study using The Health Improvement Network

**DOI:** 10.1186/s12885-018-4265-1

**Published:** 2018-04-10

**Authors:** Lucía Cea Soriano, Montse Soriano-Gabarró, Luis A. García Rodríguez

**Affiliations:** 1 Spanish Centre for Pharmacoepidemiologic Research (CEIFE), Almirante 28, 28004 Madrid, Spain; 2 Department of Public Health and Maternal and Child Health, Faculty of Medicine, Complutense University of Madrid, Madrid, Spain; 3 Epidemiology, Bayer AG, 13353 Berlin, Germany

**Keywords:** Colorectal cancer, Incidence, United Kingdom, Comorbidities

## Abstract

**Background:**

Cancer registry data show that survival of colorectal cancer (CRC) in the United Kingdom is poor compared with other European countries and the United States, yet these data sources lack information on patient comorbidities and medication use, which could help explain these differences.

**Methods:**

Among individuals aged 40–89 years in The Health Improvement Network (2000–2014), we identified first ever cases of CRC and calculated incidence rates with 95% confidence intervals (CIs). For CRC cases and non-cases in two separate calendar years (2002 and 2014), we evaluated patient demographics, lifestyle factors, comorbidities and medication use and bowel screening.

**Results:**

The incidence of CRC remained relatively constant across the study period; incidence rates per 10,000 person-years (95% CIs) were 9.27 (8.59–1.01) in 2000, 10.65 (10.15–11.18) in 2007 and 8.37 (7.93–8.83) in 2014. Incidence rates per 10,000 person-years were higher in men than women at 11.44 (95% CI: 10.35-12.66) vs. 7.40 (95% CI: 6.59–8.32) in 2000, and 9.39 (95% CI: 8.74–10.10) vs. 7.38 (95% CI: 6.81–8.00) in 2014. An increase was seen in the proportion of CRC cases diagnosed at age < 60 years. In 2002, 3.5% of CRC cases were diagnosed at age 40–49 compared with 5.1% in 2014 (*p* = 0.064). Similarly, in 2002, 12.5% were diagnosed at age 50–59 years compared with 16.2% in 2014 (*p* = 0.002). Between 2002 and 2014, previous bowel screening increased in both CRC cases (+ 10.6%) and non-cases (+ 9.7%)(*p* < 0.001 for both groups). Greater rises in the following were seen among CRC cases compared with non-cases: diabetes (+ 9.3% vs. + 3.3%; *p* < 0.001 for both), obesity (+ 14.5% vs. + 10.1%; *p* < 0.001 for both), hypertension (+ 8.3% vs. + 3.6%; *p* < 0.001 for both), atrial fibrillation (+ 2.6% [*p* < 0.01] vs. + 0.3% [*p* < 0.001]), and use of proton pump inhibitors (+ 11.5% vs. + 9.0%), anti-hypertensives (+ 9.9% vs. + 1.4%) and warfarin (+ 3.2% vs. + 0.4%); *p* < 0.001 for CRC cases and non-cases with respect to each medication.

**Conclusions:**

CRC incidence has remained relatively stable in the UK over the last decade. The increased prevalence of some comorbidities and medications among CRC cases should be considered when evaluating patterns in CRC survival.

**Electronic supplementary material:**

The online version of this article (10.1186/s12885-018-4265-1) contains supplementary material, which is available to authorized users.

## Background

In Europe, colorectal cancer (CRC) is the third most commonly diagnosed cancer in males and the second most commonly diagnosed cancer in females [1]. In the United Kingdom (UK), approximately 100 new cases of CRC are diagnosed each day [2]. Cancer registry data show that CRC incidence rates in the UK have remained relatively stable for over a decade [3] yet improvement in survival has been small [4]. Furthermore, survival rates in the UK have been poor compared with most other countries in Western and Central Europe [5, 6], Scandinavia [5, 6] and the US [7] with a relatively higher number of excess deaths in the first few months following diagnosis [8, 9] particularly among older age groups [9, 10].

While cancer registries are suitable for determining population-level incidence and survival – including adjustment for age, stage at diagnosis and socio-economic status – data such as the prevalence of comorbidities, medication use before cancer onset and other potential confounders are not systematically recorded. This hinders adjustment for these factors and limits the robustness of comparisons that can be made when making comparisons of CRC survival between populations. In the UK, prospectively collected data on patient comorbidities, prescribed medications and lifestyle factors can be ascertained from primary care databases of electronic medical records (EMRs). Using a validated UK primary care database, we conducted a retrospective cohort study to evaluate trends in the contemporary incidence of CRC in the UK and characteristics preceding CRC diagnosis, including specific comorbidities and medication use. The study protocol was approved by an independent scientific review committee (reference number 14-088A1).

## Methods

### Data source

We used data from The Health Improvement Network (THIN), a primary care database of anonymized EMRs in the UK covering approximately 6% of the UK population [11]. The database is representative of the UK population with regards to age, sex and geographic distribution, and has been validated for use in pharmacoepidemiologic research [12, 13]. Further details describing THIN can been found in the Additional file 1.

### Study population and CRC case identification

Annually, from 2000 to 2014, we identified all individuals in THIN aged 40–89 years with a registration status of permanent or died. To enter the study, individuals were required to have no previous record of any type of cancer and at least 1 years of enrolment with their primary care practitioner (PCP). All members of the study population were followed-up from the date of entry into the study year (start date) until a first recorded diagnosis for CRC, aged 90 years, death or the end of the calendar year (annually), whichever came first. Individuals with a first recorded diagnosis for CRC during follow-up were deemed to be incident cases of CRC. No additional validation step of CRC cases, such as manual review of patient records or validation with the PCP via questionnaires, was performed because we have previously shown the recording of CRC in THIN to have a high level of validity and completeness – using linkage to hospitalization data, the positive predictive value for CRC in THIN was 97.9% (556/568) and the false negative rate was 6.1% (36/592) [14].

### Covariates

For CRC cases in two separate calendar years approximately a decade apart (2002 [*N* = 931] and 2014 [*N* = 1330]), we obtained data on patient demographics (age, sex, Townsend deprivation score and urban/rural setting), lifestyle factors, healthcare use (number of PCP visits, referrals and hospitalizations), gastrointestinal comorbidities and symptoms, bowel screening procedures (colonoscopy, sigmoidoscopy, barium enema, participation in the National Bowel Screening programme), other comorbidities (with a focus on cardiovascular conditions) and medications. Lifestyle factors and BMI were ascertained any time before the start date, using the most recent value/record. Comorbidities (including gastrointestinal symptoms and bowel screening) were ascertained within the 5 years before the start date. Healthcare use was ascertained in the year before the start date, and medication use was defined as use on the start date or within the previous 30 days. For comparison, we also ascertained these data for all non-CRC cases in 2002 (*N* = 1,126,644) and 2014 (*N* = 1,758,198). For each study year this comprised all individuals who did not have a first recorded diagnosis for CRC during follow-up.

### Statistical analysis

We calculated incidence rates of CRC with 95% confidence intervals (CIs) for each calendar year in the study period using Poisson regression, censoring at the occurrence of another type of cancer. Incidence rates were calculated as the number of first-ever cases of CRC per 10,000 person-years, for the total study population and stratified by sex and 10-year age group. For patient characteristics in 2002 and 2014, data were expressed as frequency counts and percentages and differences compared using Chi2 test for categorical variables, apart from the mean age at the start date, which was presented along with its standard deviation (SD).

## Results

**Fig. 1:**
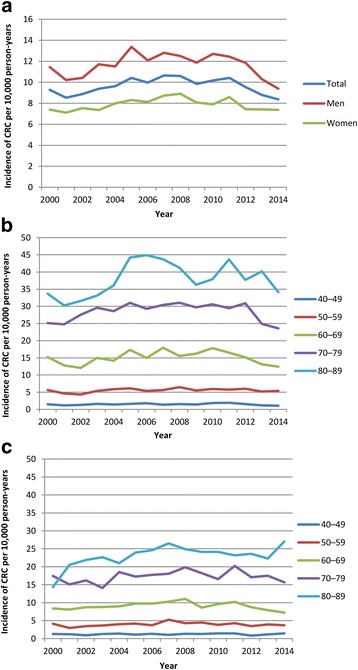
Incidence rates of colorectal cancer (CRC) per 10,000 person-years during 2000–2014 in THIN (**a**) overall and by sex, (**b**) in men by age group, and (**c**) in women by age group

The annual incidence of CRC per 10,000 person-years remained relatively constant across the study period; incidence rates increased from 9.27 (95% CI: 8.59–1.01) in 2000 to 10.65 (95% CI: 10.15–11.18) in 2007, followed by a decreased trend during the later years in the study period, falling to 8.37 (95% CI: 7.93–8.83) in 2014 (Fig. 1a). In men, the incidence of CRC per 10,000 person-years was 11.44 (95% CI: 10.35–12.66) in 2000 and 9.39 (95% CI: 8.74–10.10) in 2014; corresponding rates for women were 7.40 (95% CI: 6.59–8.32) and 7.38 (95% CI: 6.81–8.00). Incidence rates by age at diagnosis across the study period are shown in Fig. 1b for men and Fig. 1c for women. The declining incidence of CRC in the later years of the study period was mainly driven by decreasing rates in older men (aged ≥60 years) from 2012, while overall rates in women remained relatively stable during these study years. As shown in Table 1, incidence rates of CRC were of similar magnitude and followed a similar trend to those reported by the Office for National Statistics (ONS) for the UK over the same study period. Rates were slightly higher in THIN but this could be owing to the fact that our study population was among adults aged 40–89 years while the ONS rates are among all individuals.

**Table 1:** Annual incidence rates of CRC per 10,000 person-years in the UK (2000–2014): comparison of data from THIN and ONS

	Incidence rates of CRC per 10,000 person-years														
	2000	2001	2002	2003	2004	2005	2006	2007	2008	2009	2010	2011	2012	2013	2014
Total															
THIN	9.27	8.54	8.87	9.38	9.63	10.41	9.98	10.65	10.59	9.86	10.17	10.42	9.53	8.79	8.37
ONS	7.26	7.04	6.94	6.99	7.15	7.17	7.26	7.36	7.48	7.55	7.53	7.60	7.54	7.21	7.00
Males															
THIN	11.44	10.20	10.41	11.71	11.51	13.37	12.07	12.80	12.49	11.88	12.71	12.44	11.85	10.30	9.39
ONS	9.23	8.96	8.79	8.96	9.09	9.12	9.12	9.21	9.44	9.49	9.48	9.45	9.46	8.95	8.61
Female															
THIN	7.40	7.10	7.54	7.35	7.98	8.30	8.13	8.74	8.90	8.06	7.90	8.60	7.43	7.41	7.38
ONS	5.79	5.64	5.55	5.51	5.68	5.69	5.80	5.91	5.94	6.00	5.97	6.08	6.01	5.78	5.69

Age ranges were 40–89 years in THIN; all ages in ONS

*CRC* colorectal cancer, *ONS* Office for National Statistics, *THIN* The Health Improvement Network, *UK* United Kingdom

### Characteristics of incident CRC cases and non-cases in 2002 and 2014

Demographics, lifestyle factors and healthcare use among CRC cases and non-cases in 2002 and 2014 are shown in Table 2. No substantial change was seen in the proportion of male and female cases of incident CRC between the two study years (*p* = 0.711). A rise was seen in the proportion of CRC cases diagnosed at ages < 60 years; in 2002, 3.5% of CRC cases were diagnosed at age 40–49 compared with 5.1% in 2014 (*p* = 0.064). Similarly, in 2002, 12.5% were diagnosed at age 50–59 years compared with 16.2% in 2014 (*p* = 0.002). The mean age at diagnosis was 70.2 years (SD: 10.2) in 2002 and 69.4 years (SD: 11.2) in 2014. Corresponding data (age at start date) for non-cases was 58.6 (SD: 12.8) in 2002 and 57.6 (SD: 12.6) in 2014. Between 2002 and 2014, the proportion of current smokers among CRC cases decreased by 3.1%, with a similar decrease seen among non-cases (− 2.5%) (*p* < 0.001 for both). A decline in non-drinkers was seen in both cases and non-cases (*p* < 0.001). While obesity (BMI ≥30 kg/m^2^) was recorded more frequently in 2014 than in 2002 in both groups (*p* < 0.001), the increase was greater among CRC cases than for non-cases (+ 14.5% vs. + 10.1%).

**Table 2:** Demographics, lifestyle factors and healthcare use among CRC cases and non-cases in 2002 and 2014

	CRC cases				Non-cases			
	2002 (*N* = 921)	2014 (*N* = 1330)	% change	*p* value	2002 (*N* = 1,125,723)	2014 (*N* = 1,756,868)	% change	*p* value
	*n* (%)	*n* (%)			*n* (%)	*n* (%)		
Sex				0.711				< 0.001
Male	501 (54.4)	734 (55.2)	+ 0.8		523,503 (46.5)	866,071 (49.3)	+ 2.8	
Female	420 (45.6)	596 (44.8)	−0.8		602,220 (53.5)	890,797 (50.7)	−2.8	
Age at start date^a^ (years)				0.001				< 0.001
40–49	32 (3.5)	68 (5.1)	+ 1.6		330,063 (29.3)	577,167 (32.9)	+ 3.6	
50–59	115 (12.5)	215 (16.2)	+ 3.7		305,523 (27.1)	464,427 (26.4)	−0.7	
60–69	244 (26.5)	334 (25.1)	−1.4		234,955 (20.9)	374,036 (21.3)	+ 0.4	
70–79	355 (38.5)	421 (31.7)	−6.8		174,098 (15.5)	229,621 (13.1)	−2.4	
80–89	175 (19.0)	292 (22.0)	+ 3.0		81,084 (7.2)	111,617 (6.4)	−0.8	
Smoking				< 0.001				< 0.001
Non-smoker	393 (42.7)	575 (43.2)	+ 0.5		511,656 (45.5)	841,369 (47.9)	+ 2.4	
Current	145 (15.7)	168 (12.6)	−3.1		229,660 (20.4)	314,311 (17.9)	−2.5	
Former	230 (25.0)	580 (43.6)	+ 18.6		190,297 (16.9)	577,415 (32.9)	+ 16	
Unknown	153 (16.6)	7 (0.5)	−16.1		194,110 (17.2)	23,773 (1.4)	−15.8	
BMI (kg/m^2^)				< 0.001				< 0.001
15–19	23 (2.5)	48 (3.6)	+ 1.1		39,966 (3.6)	65,177 (3.7)	+ 0.1	
20–24	253 (27.5)	316 (23.8)	−3.7		316,584 (28.1)	482,255 (27.4)	−0.7	
25–29	298 (32.4)	517 (38.9)	+ 6.5		323,628 (28.7)	594,699 (33.8)	+ 5.1	
≥ 30	116 (12.6)	360 (27.1)	+ 14.5		169,036 (15.0)	441,310 (25.1)	+ 10.1	
Unknown	231 (25.1)	89 (6.7)	−18.4		276,509 (24.6)	173,427 (9.9)	−14.7	
Polypharmacy				< 0.001				< 0.001
0–1 medications	517 (56.1)	612 (46.0)	−10.1		792,376 (70.4)	1,151,402 (65.5)	−4.9	
2–4 medications	273 (29.6)	357 (26.8)	−2.8		231,408 (20.6)	345,485 (19.7)	−0.9	
≥ 5 medications	131 (14.2)	361 (27.1)	+ 12.9		101,939 (9.1)	259,981 (14.8)	+ 5.7	
Alcohol (u/w)				< 0.001				< 0.001
None	113 (12.3)	230 (17.3)	+ 5.0		150,487 (13.4)	268,029 (15.3)	+ 1.9	
1–9	392 (42.6)	621 (46.7)	+4.1		471,097 (41.8)	785,666 (44.7)	+ 2.9	
10–20	116 (12.6)	219 (16.5)	+ 3.9		142,656 (12.7)	297,466 (16.9)	+ 4.2	
21–41	45 (4.9)	72 (5.4)	+ 0.5		48,314 (4.3)	94,073 (5.4)	+ 1.1	
≥ 42	15 (1.6)	31 (2.3)	+ 0.7		16,227 (1.4)	40,976 (2.3)	+ 0.9	
Unknown	240 (26.1)	157 (11.8)	−14.3		296,942 (26.4)	270,658 (15.4)	−11	
PCP visits^b^				< 0.001				< 0.001
0–4	374 (40.6)	249 (18.7)	−21.9		622,029 (55.3)	582,007 (33.1)	−22.2	
5–9	252 (27.4)	319 (24.0)	−3.4		277,673 (24.7)	435,570 (24.8)	+ 0.1	
10–14	135 (14.7)	242 (18.2)	+ 3.5		121,781 (10.8)	291,525 (16.6)	+ 5.8	
15–19	79 (8.6)	171 (12.9)	+4.3		54,652 (4.9)	177,759 (10.1)	+ 5.2	
≥ 20	81 (8.8)	349 (26.2)	+ 17.4		49,588 (4.4)	270,007 (15.4)	+ 11	
Referrals^b^				< 0.001				< 0.001
0–1	636 (69.1)	531 (39.9)	−29.2		920,723 (81.8)	929,757 (52.9)	−28.9	
2–4	218 (23.7)	380 (28.6)	+4.9		155,781 (13.8)	464,696 (26.5)	+ 12.7	
5–9	58 (6.3)	279 (21.0)	+ 14.7		41,329 (3.7)	261,011 (14.9)	+ 11.2	
≥ 10	9 (1.0)	140 (10.5)	+ 9.5		7890 (0.7)	101,404 (5.8)	+ 5.1	
Hospitalizations^b^				< 0.001				< 0.001
None	829 (90.0)	1109 (83.4)	−6.6		1,063,618 (94.5)	1,557,111 (88.6)	−5.9	
1	57 (6.2)	133 (10.0)	+ 3.8		43,745 (3.9)	134,083 (7.6)	+ 3.7	
2	23 (2.5)	49 (3.7)	+ 1.2		11,974 (1.1)	40,510 (2.3)	+ 1.2	
≥ 3	12 (1.3)	39 (2.9)	+ 1.6		6386 (0.6)	25,164 (1.4)	+ 0.8	
Townsend score				< 0.001				< 0.001
Quintile 1 (least deprived)	225 (24.4)	340 (25.6)	+ 1.2		304,177 (27.0)	466,028 (26.5)	−0.5	
Quintile 2	183 (19.9)	329 (24.7)	+4.8		253,931 (22.6)	394,091 (22.4)	−0.2	
Quintile 3	180 (19.5)	297 (22.3)	+ 2.8		219,425 (19.5)	355,598 (20.2)	+ 0.7	
Quintile 4	183 (19.9)	185 (13.9)	−6.0		178,640 (15.9)	289,718 (16.5)	+ 0.6	
Quintile 5 (most deprived)	98 (10.6)	140 (10.5)	−0.1		117,993 (10.5)	191,274 (10.9)	+ 0.4	
Unknown	52 (5.6)	39 (2.9)	−2.7	0.001	51,557 (4.6)	60,159 (3.4)	−1.2	< 0.001
Setting								
Urban	562 (61.0)	748 (56.2)	−4.8		720,972 (64.0)	1,086,412(61.8)	−2.2	
Town	120 (13.0)	148(11.1)	−1.9		129,017 (11.5)	178,340 (10.2)	−1.3	
Rural	58 (6.3)	73 (5.5)	−0.8		76,435 (6.8)	98,160 (5.6)	−1.2	
Unknown	181 (19.7)	361 (27.1)	+ 7.4		199,299 (17.7)	393,956 (22.4)	+4.7	

^a^Entry into the study year (start date)

^b^In the year before the start date

*BMI* body mass index, *CRC* colorectal cancer, *PCP* primary care practitioner, *u/w* units per week

Gastrointestinal comorbidities, symptoms and investigative procedures among CRC cases and non-cases in 2002 and 2014 are shown in Table 3. As expected, an increase was seen in the number of individuals with a prior record of participation in the National Bowel Screening programme (+ 10.6% in CRC cases, *p* < 0.001 and + 9.7% in non-cases, *p* < 0.001). The proportion of cases with a record of colonoscopy also increased among CRC cases (2.8% to 6.2%, *p* < 0.001) and in non-cases (1.6% to 4.2%, *p* < 0.001). The prevalence of sigmoidoscopy was similar across the study years in cases (*p* = 0.412) but increased among non-cases (*p* < 0.001), while the prevalence of barium enema decreased among both groups (*p* < 0.001 for both).

**Table 3:** Gastrointestinal comorbidities, symptoms and investigative procedures among CRC cases and non-cases in 2002 and 2014

	CRC cases			*p*-value	Non-cases			*p*-value
	2002 (*N* = 921)	2014 (*N* = 1330)	% change		2002 (*N* = 1,125,723)	2014 (*N* = 1,756,868)	% change	
	*n* (%)	*n* (%)			*n* (%)	*n* (%)		
Bleeding per rectum	79 (8.6)	73 (5.5)	− 3.1	0.004	22,989 (2.0)	38,394 (2.2)	+ 0.2	< 0.001
Change in bowel habits	26 (2.8)	54 (4.1)	+ 1.3	0.119	12,190 (1.1)	29,360 (1.7)	+ 0.6	< 0.001
Abnormal weight loss	19 (2.1)	26 (2.0)	−0.1	0.857	7972 (0.7)	20,924 (1.2)	+ 0.5	< 0.001
GI adenoma	20 (2.2)	41 (3.1)	+ 0.9	0.191	5035 (0.4)	16,565 (0.9)	+ 0.5	< 0.001
GORD	48 (5.2)	88 (6.6)	+ 1.4	0.169	55,237 (4.9)	110,752 (6.3)	+ 1.4	< 0.001
Complicated/uncomplicated PU	20 (2.2)	28 (2.1)	−0.1	0.915	13,891 (1.2)	13,544 (0.8)	−0.4	< 0.001
Complicated PU	9 (1.0)	23 (1.7)	+ 0.7	0.138	6994 (0.6)	9015 (0.5)	−0.1	< 0.001
Uncomplicated PU	13 (1.4)	8 (0.6)	−0.8	0.049	7977 (0.7)	5484 (0.3)	−0.4	< 0.001
IBD	19 (2.1)	25 (1.9)	−0.2	0.700	26,919 (2.4)	33,242 (1.9)	−0.5	0.032
National bowel screening programme	34 (3.7)	190 (14.3)	+ 10.6	< 0.001	< 0.001	187,548 (10.7)	+ 9.7	< 0.001
Colonoscopy	26 (2.8)	83 (6.2)	+ 3.4	< 0.001	17,952 (1.6)	73,186 (4.2)	+ 2.6	< 0.001
Sigmoidoscopy	44 (4.8)	54 (4.1)	−0.7	0.412	19,980 (1.8)	36,673 (2.1)	+ 0.3	< 0.001
Barium enema	95 (10.3)	14 (1.1)	−9.2	< 0.001	45,149 (4.0)	9619 (0.5)	−3.5	< 0.001

*CRC* colorectal cancer, *GI* gastrointestinal, *GORD* gastro-oesophageal reflux disease, *IBD* irritable bowel disease, *PU* peptic ulcer

As shown in Table 4, the prevalence of diabetes, hypertension, atrial fibrillation increased between the two study years in both CRC cases and non-cases, but with a greater increase seen in cases: + 8.8% (*p* < 0.001) vs. + 6.5% (*p* < 0.001) for diabetes, + 8.3% (*p* < 0.001) vs. + 3.6% (*p* < 0.001) for hypertension, and + 2.6% (*p* < 0.01) vs. + 0.3% (*p* < 0.001) for atrial fibrillation. This trend was also seen with use of proton pump inhibitors (PPIs), anti-hypertensives and warfarin use at the start date (*p* < 0.001 for all) (Table 5). Current use of low-dose aspirin increased slightly in CRC cases (*p* < 0.001) and decreased slightly in non-cases (*p* < 0.001), while current use of non-steroidal anti-inflammatory drugs decreased in both CRC cases (*p* = 0.002) and non-cases (*p* < 0.001). Statin prescribing notably increased among cases (+ 26.8%, *p* < 0.001) and increased but to a lesser extent among non-cases (+ 13.0%, *p* < 0.001).

**Table 4:** Distribution of cardiovascular and other comorbidities among CRC cases and non-cases in 2002 and 2014

	CRC cases			*p*-value	Non-cases			*p*-value
	2002 (*N* = 921)	2014 (*N* = 1330)	% change		2002 (*N* = 1,125,723)	2014 (*N* = 1,756,868)	% change	
	*n* (%)	*n* (%)			*n* (%)	*n* (%)		
IHD	92 (10.0)	74 (5.6)	−4.4	< 0.001	73,352 (6.5)	48,806 (2.8)	−3.7	< 0.001
Hypertension	225 (24.4)	435 (32.7)	+ 8.3	< 0.001	176,647 (15.7)	339,158 (19.3)	+ 3.6	< 0.001
Hypercholesterolaemia	35 (3.8)	70 (5.3)	+ 1.5	0.106	37,220 (3.3)	82,270 (4.7)	+ 1.4	< 0.001
DVT/PE	33 (3.6)	37 (2.8)	−0.8	0.282	20,408 (1.8)	27,139 (1.5)	−0.3	< 0.001
Heart failure	30 (3.3)	30 (2.3)	−1.0	0.147	17,086 (1.5)	16,112 (0.9)	−0.6	< 0.001
Atrial fibrillation	33 (3.6)	82 (6.2)	+ 2.6	0.006	20,783 (1.8)	36,865 (2.1)	+ 0.3	< 0.001
MI	16 (1.7)	25 (1.9)	+ 0.2	0.804	15,651 (1.4)	17,206 (1.0)	−0.4	< 0.001
Ischaemic stroke	12 (1.3)	20 (1.5)	+ 0.2	0.692	12,953 (1.2)	17,983 (1.0)	−0.2	< 0.001
TIA	15 (1.6)	23 (1.7)	+ 0.1	0.855	12,642 (1.1)	15,200 (0.9)	−0.2	< 0.001
Haemorrhagic stroke	3 (0.3)	5 (0.4)	+ 0.1	0.844	1418 (0.1)	2370 (0.1)	0.0	0.041
Anaemia	53 (5.8)	77 (5.8)	0.0	0.972	24,250 (2.2)	47,283 (2.7)	+ 0.5	< 0.001
Diabetes	79 (8.6)	231 (17.4)	+ 8.8	< 0.001	60,930 (5.4)	155,718 (8.9)	+ 6.5	< 0.001
Depression	66 (7.2)	86 (6.5)	−0.7	0.515	106,405 (9.5)	171,204 (9.7)	+ 0.2	< 0.001
COPD	44 (4.8)	93 (7.0)	+ 2.2	0.031	24,696 (2.2)	64,071 (3.6)	+ 1.4	< 0.001
Asthma	92 (10.0)	157 (11.8)	+ 1.8	0.177	78,615 (7.0)	199,049 (11.3)	+4.3	< 0.001
Osteoarthritis	146 (15.9)	196 (14.7)	−1.2	0.468	123,356 (11.0)	157,758 (9.0)	−2.0	< 0.001
Rheumatoid arthritis	13 (1.4)	14 (1.1)	−0.3	0.442	10,383 (0.9)	18,234 (1.0)	+ 0.1	< 0.001

*CRC* colorectal cancer, *COPD* chronic obstructive pulmonary disease, *DVT* deep vein thrombosis, *IHD* ischaemic heart disease, *MI* myocardial infarction, *PE* pulmonary embolism, *TIA* transient ischaemic attack

**Table 5:** Principal drug therapies prescribed among CRC cases and non-cases in 2002 and 2014

	CRC cases			*p*-value	Non-cases			*p*-value
	2002 (*N* = 921)	2014 (*N* = 1330)	% change		2002 (*N* = 1,125,723)	2014 (*N* = 921)	% change	
Low-dose aspirin	150 (16.3)	220 (16.5)	+ 0.2	< 0.001	113,852 (10.1)	156,206 (8.9)	−1.2	< 0.001
Clopidogrel	7 (0.8)	40 (3.0)	+ 2.2	< 0.001	4441 (0.4)	30,405 (1.7)	+ 1.3	< 0.001
Dipyridamole	8 (0.9)	7 (0.5)	−0.4	0.326	3787 (0.3)	4040 (0.2)	−0.1	< 0.001
Warfarin	29 (3.1)	84 (6.3)	+ 3.2	0.001	19,844 (1.8)	37,797 (2.2)	+ 0.4	< 0.001
All NSAIDs	68 (7.4)	57 (4.3)	−3.1	0.002	92,727 (8.2)	97,302 (5.5)	−2.7	< 0.001
tNSAIDs	60 (6.5)	52 (3.9)	−2.6	0.005	79,946 (7.1)	91,889 (5.2)	−1.9	< 0.001
Coxibs	8 (0.9)	6 (0.5)	−0.4	0.215	13,827 (1.2)	5655 (0.3)	−0.9	< 0.001
Insulin	15 (1.6)	40 (3.0)	+ 1.4	0.037	12,532 (1.1)	27,067 (1.5)	+ 0.4	< 0.001
Oral antidiabetics	36 (3.9)	150 (11.3)	+ 7.4	< 0.001	35,496 (3.2)	101,716 (5.8)	+ 2.6	< 0.001
Oral corticosteroids	23 (2.5)	35 (2.6)	+ 0.1	0.843	20,602 (1.8)	35,869 (2.0)	+ 0.2	< 0.001
Inhaled steroids	66 (7.2)	74 (5.6)	−1.6	0.0122	54,881 (4.9)	78,009 (4.4)	−0.5	< 0.001
Statins	95 (10.3)	493 (37.1)	+ 26.8	< 0.001	82,486 (7.3)	355,803 (20.3)	+ 13.0	< 0.001
Non-statins	9 (1.0)	7 (0.5)	−0.5	0.211	5192 (0.5)	7766 (0.4)	−0.1	0.018
Antidiarrhoeal medications	29 (3.1)	42 (3.2)	+ 0.1	0.990	13,101 (1.2)	32,378 (1.8)	+ 0.6	< 0.001
Antidepressants	69 (7.5)	149 (11.2)	+ 3.7	0.003	92,058 (8.2)	217,632 (12.4)	+4.2	< 0.001
Acetaminophen	150 (16.3)	249 (18.7)	+ 2.4	0.137	123,387 (11.0)	211,478 (12.0)	+ 1.0	< 0.001
PPIs	97 (10.5)	293 (22.0)	+ 11.5	< 0.001	69,225 (6.1)	266,005 (15.1)	+ 9.0	< 0.001
Antihypertensive medications	409 (44.4)	722 (54.3)	+ 9.9	< 0.001	316,728 (28.1)	519,072 (29.5)	+ 1.4	< 0.001

*Coxibs* COX-2-selective inhibitors, *CRC* colorectal cancer, *NSAIDs* non-steroidal anti-inflammatory drugs, *PPIs* proton pump inhibitors, *tNSAIDs* traditional non-steroidal anti-inflammatory drugs

## Discussion

In this large population-based study set in a representative primary care setting, we have described the contemporary epidemiology of CRC in the UK and characterized CRC patients at the time of diagnosis in two calendar years more than a decade apart. Few studies have described changes in the comorbidity profile of CRC patients over time [15] and we are unaware of any other study to describe a wide range of patient comorbidities and medication use.

While we have shown that the incidence of CRC has remained relatively stable in the UK over the last 15 years, incidence rates appear to have declined in more recent years, particularly in men aged ≥60 years. Our study also found increased uptake of the National Bowel Screening programme over the study period, which could explain the finding of a slightly earlier mean age at diagnosis in 2014 compared with 2002. The national faecal occult blood test screening programme was rolled out in England in June 2006 and in Scotland in June 2007 with the aim of reducing the number of incident cases through detecting pre-cancerous CRC adenomas. Primary care practitioners involved in the programme were informed of participants who failed to complete the programme, and this may have contributed to an increase in the detection or reporting of new cases that may have previously gone unnoticed – this could explain the peak in CRC incidence rates in this current study in 2007. The incidence rates of CRC in this present study are in line, albeit slightly higher, with those from UK Cancer Registry data [3] and similar age- and gender-specific trends were seen over the study period.

Several factors should ideally be taken into account when analyzing trends in survival of patients with CRC across time and when making comparisons with other geographical populations, including the comorbidity profile and medication use of patients at the time of diagnosis. Several studies have shown that high comorbidity levels are associated with poorer survival [10, 16–22]. In the National Cancer Data Repository, Downing et al. [10] showed that 24.2% of colon cancer patients who died within the first month following diagnosis had a Charlson comorbidity score of ≥3, compared with 17.6%, 14.2% and 7.2% with a score of two, one or zero, respectively. In a population-based study in Denmark, Erichsen et al. [23] showed that comorbidities interacted with CRC to increase mortality beyond that explained by CRC and comorbidities acting independently, particularly in the first year after CRC diagnosis. In the Netherlands, overall comorbidity among over 27,000 CRC patients in the Eindhoven cancer registry increased from 47% to 62% between 1995 and 2010, with hypertension increasing from 16% to 29% and cardiovascular disease increasing from 12% to 24% [15]. Diabetes, hypertension and atrial fibrillation and obesity, were the main characteristics observed to be more prevalent among CRC cases diagnosed in 2014 than among those diagnosed in 2002. Although these conditions were also more prevalent among non-cases in these two study years, the level of increase was notably higher among CRC cases, especially for diabetes. It is possible that more frequent PCP visits among patients presenting with CRC symptoms, or a higher level of monitoring among high-risk patients, could have led to better recording of other comorbidities and lifestyle factors. However, a genuine greater increase in these conditions among CRC patients could translate into more CRC patients having fewer treatment options and ultimately worse survival. In a large study of older age CRC patients investigating population-attributable risks, a substantial proportion of deaths were attributable to congestive heart failure, diabetes and chronic obstructive pulmonary disease [22]. In our study, a 8.8% increase in the prevalence of diabetes at the time of CRC diagnosis was observed between study years, although only minor changes in the prevalence of heart failure and chronic obstructive pulmonary disease were seen. In relation to medication use at the time of diagnosis, higher increases in the use of PPIs, anti-hypertensives and warfarin among CRC cases compared with non-cases between 2002 and 2014 suggests the growing pill burden among CRC patients, a factor that may also influence treatment options in addition to adherence and outcomes. With an aging general population, non-cancer related health status could play an increasingly important role in the survival of cancer patients, highlighting the importance of data such as those obtained in this study.

Strengths of our study include the large sample size representative of the UK population as a whole; Blak et al. [13] have shown both the demographic of individuals and the prevalence of major medical conditions in THIN to be generalisable to the UK. In addition, previous validation work has shown a high level of completeness and validity of the recorded CRC diagnoses in THIN [14]. Although we did not link to cancer registry data in our prior validation study [14] another study comparing incidence rates in THIN with those in a UK national cancer registry found the age- and sex-standardized incidence rates of CRC to be very similar during the latter part of their study period (2005–2007) [24]. In addition, a study using data from the UK Clinical Practice Research Datalink, which contains very similar primary care data to THIN, reported a 98% PPV for the CRC diagnosis in the primary care data when linked to cancer registrations [25]. A limitation of our study is the possible improvement in recording in THIN over time, resulting in an under-estimation of CRC incidence rates and patient characteristics in the early years of the study period. Indeed, there was a notable improvement in the proportion of both CRC cases and non-cases with a recorded BMI, smoking status or alcohol intake between 2002 and 2014. Prior to 2003, PCPs contributing data to THIN did not have specific recording instructions, although any improvements in recording will likely have affected CRC cases and non-cases equally. The year 2003 also saw the introduction of a cancer quality improvement measures in the UK by the NHS, which may not only have improved recording of CRC diagnoses but also of other morbidities and patient characteristics. This would have led to a more complete profile in the patient’s records.

Advanced stage at CRC diagnosis is clearly associated with worse survival [26, 27], and accounts for the highest proportion of early colon cancer deaths; Downing et al. [10] reported 15.4% of colon cancer deaths in the month following diagnosis to be Duke’s Stage D, compared with approximately 5% or less for less advanced stages of the disease. There was insufficient information in our dataset to enable us to ascertain Duke’s stage of CRC cases at diagnosis and thereby describe any potential changes over time in the proportion of CRC cases diagnosed at advanced stage. Failing to reduce the proportion of CRC cases diagnosed at advanced stage compared with other countries could be a possible explanation for the lower relative survival rates and small improvements in survival rates over the last decade. Diagnostic codes in THIN are not specific for Duke’s stage CRC; rather information on CRC histology and stage is entered into patients’ medical notes as free-text comments, which were not obtained in this study. We have, however, previously evaluated CRC stage at diagnosis in another study in THIN [28], which included 3033 incident cases of CRC diagnosed between 2000 and 2011 with Duke’s stage ascertained through manual review of patients EMRs including the free-text comments. We found that Duke’s stage D accounted for 12.7% of CRC cases diagnosed in 2002 and 16.3% of CRC cases in 2011, whereas reductions were seen in the proportion of CRC cases diagnosed at Duke’s stages A to C between these two calendar years (6.7% to 4.1% for Duke’s stage A; 14.4% to 9.2% for Duke’s stage B; 11.0% to 8.9% for Duke’s stage C; *unpublished data*). It should be noted that 55.1% of the CRC cases in 2002 and 61.4% of CRC cases in 2011 did not have details on stage recorded in the free text.

## Conclusion

In conclusion, our findings suggest that the increased prevalence of obesity, hypertension, atrial fibrillation and the pill burden among CRC cases should be considered when evaluating and making comparisons in patterns of CRC survival.

## Additional file

Additional file 1: Supplementary Methods. (DOCX 13 kb)

## Abbreviations

CI: Confidence interval
EMR: Electronic medical records
PCP: Primary care practitioner
THIN: The Health Improvement Network
UK: United Kingdom
