# Supplementary material for: Trends in the contemporary incidence of colorectal cancer and patient characteristics in the United Kingdom: a population-based cohort study using The Health Improvement Network
Source: BMC Cancer. 2018 Apr 10;18:402. doi: 10.1186/s12885-018-4265-1 (PMC5894203; doi:10.1186/s12885-018-4265-1)
Supplement: Supplementary file 1 — Supplementary Methods. (DOCX 13 kb) [file 12885_2018_4265_MOESM1_ESM.docx]

**Additional file 1**

Almost 600 general practices throughout the UK contribute data to THIN (1). Participating PCPs record data prospectively as part of their routine patient care, and send their data anonymously to THIN for use in research projects. Patient information is entered using Read codes or as free text. Read codes are the standard clinical terminology used in UK general practice, supporting detailed clinical encoding of diagnoses, symptoms, laboratory tests and results, therapeutics, surgical procedures and demographics (2). Prescriptions are entered using Gemscript codes based on the National Health Service’s (NHS) dictionary of medicines and devices (3) and are automatically recorded upon issue. Information from secondary care is communicated back to the PCP and entered in the database retrospectively.

**References**

1. IMS Health. THIN data. Statistics. MS Health. THIN data. Statistics. http://www.csdmruk.imshealth.com/our-data/statistics.shtml. Accessed 24 March 2017.

2. Stuart-Buttle CD, Read JD, Sanderson HF, Sutton YM. A language of health in action: Read Codes, classifications and groupings. Proc AMIA Annu Fall Symp 1996:75–9.

3. In Practice Systems Ltd. Gemscript Â The new DM+D drug dictionary for Vision. In Practice Systems Ltd Gemscript Â The new DM+D drug dictionary for Vision. http://www.inps4.co.uk/my-vision/news/gemscript-%C3%82%C2%96-new-dmd-drug-dictionary-vision. Accessed 24 March 2017.
